# Association of the prognostic model iSEND with PD-1/L1 monotherapy outcome in non-small-cell lung cancer

**DOI:** 10.1038/s41416-019-0643-y

**Published:** 2019-11-25

**Authors:** Wungki Park, Laura Mezquita, Naoyuki Okabe, Young Kwang Chae, Deukwoo Kwon, Diana Saravia, Edouard Auclin, David Planchard, Caroline Caramella, Roberto Ferrara, Sarita Agte, Michael Oh, Raja Mudad, Mohammad Jahanzeb, Hiroyuki Suzuki, Benjamin Besse, Gilberto Lopes

**Affiliations:** 1 University of Miami, Miller School of Medicine, Sylvester Comprehensive Cancer Center, Miami, FL USA; 2 Memorial Sloan Kettering Cancer Center, New York, NY USA; 3 Weill Cornell Medical College, New York, NY USA; 4 Gustave Roussy, Medical Oncology Department, Thoracic Oncology Group, Villejuif, France; 5 Department of Chest Surgery, Fukushima Medical University, School of Medicine, Fukushima, Japan; 6 Department of Medicine, Northwestern University Feinberg School of Medicine, Chicago, IL USA

**Keywords:** Non-small-cell lung cancer, Prognostic markers

## Abstract

**Background:**

Accessible biomarkers are needed for immunotherapy in advanced non-small-cell lung cancer (NSCLC). We previously described a multivariate risk prediction model, the iSEND, which categorises advanced NSCLC patients treated with nivolumab into Good, Intermediate or Poor groups. This model was developed by using only clinical and analytical variables (sex, ECOG-performance status, neutrophil-to-lymphocyte ratio [NLR] and post-treatment delta NLR).

**Methods:**

An international database of 439 patients who received post-platinum PD-1/L1 monotherapies was collected for validation. Performance of the iSEND to different PD-L1 groups was compared by using time-dependent positive predictive value (PPV) for their mortality events.

**Results:**

Median follow-up was 18.2 months (95% CI: 15.9–19.6). The overall survival of the iSEND Good (HR = 0.31, 95% CI: 0.22–0.43, *p* < 0.0001) was superior to the iSEND Poor. Time-dependent PPV for mortality of iSEND Poor was superior to PD-L1 = 0% group at 12 (75 vs. 53%, *p* = 0.01) and 18 months (85 vs. 46%, *p* = 0.03). However, female gender did not independently associate with better outcome in the validation cohort.

**Conclusion:**

The iSEND model is associated with the outcome of post-platinum PD-1/L1 monotherapy in advanced NSCLC patients. The iSEND Poor demonstrated a superior performance to PD-L1 = 0% in negative prognostication. Prospective investigation and modelling with other significant parameters in a larger cohort are warranted.

## Background

Immunotherapy has become an invaluable treatment option for cancer patients.^1–7^ The programmed death-1/-ligand 1 monotherapies have set out the milestones in oncology with superior patient survival and tolerance over conventional chemotherapy in advanced non-small-cell lung cancers (NSCLC).^1,2,4–6^ Nivolumab and atezolizumab were approved by Food Drug Administration (FDA) and European Medical Agency without PD-L1 immunohistochemistry level in the setting of second line and above, and pembrolizumab was approved in patients with PD-L1 ≥ 50% in the first-line setting as well as PD-L1 ≥ 1% in above first line.^1,2,6,7^ Likewise, clinical indications for combinational immunotherapy by using the backbone of PD-1/L1 monotherapy and cytotoxic-T-lymphocyte-associated protein-4 (CTLA-4) inhibitor continue to expand.^5,8–10^ Nevertheless, the lack of optimal biomarker leaves the biggest challenge unresolved in the field.

Major effort in biomarker research has focused on biomarker development to best select individuals who can best benefit from the immunotherapy. Currently, PD-L1 is the most validated biomarker in NSCLC patients treated with PD-1/L1 monotherapy.^6,11,12^ However, there are controversies regarding different assay antibodies and variable cut-offs. In parallel, different novel biomarker strategies have been developed, including histology-agnostic biomarkers for immunotherapy like tumour mutational burden (TMB) currently under investigation and microsatellite instability status, which was already approved by FDA as a predictive biomarker for PD-1/L1 monotherapy.^9,13–16^ These agnostic markers have advanced the field rapidly, and we are learning more about the biology of cancer immunotherapy. But discrepancy of available resources and high cost continue to be the hurdle for the use of immunotherapy in different parts of the globe. Conceivably on the other hand, highly accessible, inexpensive and noninvasive biomarkers like peripheral blood parameters such as neutrophil-to-lymphocyte ratio (NLR) and derived neutrophil-to-lymphocyte ratio have been suggested as practical markers for immunotherapy.^17–21^

Neutrophil-to-lymphocyte ratio (NLR) is an inexpensive, universally accessible and minimally invasive method that can be obtained by a simple peripheral blood draw.^18,20^ Many clinical studies support the negative prognostic value of high NLR in immunotherapy.^18,22–24^ High NLR has been suggested to represent the immunosuppressive phenotypes from circulating neutrophils more than the lymphocytes. Moreover, the reactive increase of neutrophils in tumour microenvironment in immunotherapy-resistant patients as suppressive myeloid cells was suggested as a resistance mechanism, which was modelled as positive delta NLR (DNLR).^25–27^ The on-treatment increase of neutrophil in peripheral blood in human and mouse models has shown the resistance in multiple preclinical immunotherapy models, and reversing high NLR to low NLR with c-MET inhibition showed enhanced immunotherapy outcome in mice.^25^

Our group previously reported the development of multivariate risk prediction model called the iSEND model (immunotherapy Sex, ECOG, NLR [Neutrophil-to-Lymphocyte Ratio], Delta NLR).^27^ The model was developed from retrospective analysis by using aforementioned variables of the advanced NSCLC patients from the University of Miami treated with post-platinum nivolumab monotherapy. The iSEND model was designed to reflect the highest weighting coefficient from the composite score of NLR and DNLR to better identify more resistant patients to nivolumab, which identifies the patients with high baseline NLR and its subsequent increase after the first dose of nivolumab. Here, we show the independent validation as well as pooled cohort analysis, which were compared with chemotherapy-only-treated cohort. Also, we show performance comparison of the iSEND groups with different PD-L1 expression-level groups to discuss potential clinical implications of the iSEND model in advanced NSCLC patients treated with PD-1/L1 monotherapy.

## Methods

### Study population

A retrospective database of four participating institutions was collected for pathologically confirmed diagnosis of advanced NSCLC patients treated with PD-1/L1 monotherapy, including nivolumab, pembrolizumab or atezolizumab, in the post-platinum setting from March 2015 to December 2017. An additional independent cohort of NSCLC patients who received second-line chemotherapy without immunotherapy was collected for comparison from the University of Miami. The Local Institutional Review Board approved for this study at each participating institution in accordance with the tenets of the Declaration of Helsinki. PD-1/L1 monotherapy treatment was administered until progression, unacceptable toxicity or death according to dosing instructions provided from each agent’s manufacturer. Patients usually underwent radiographic imaging every 8–12 weeks for response evaluation by computed tomography.

### PD-L1 cut-offs and groups

Patients with PD-L1 expression available were selected for performance comparison with iSEND Good or Poor groups for mortality (OS) and progression (PFS) prediction by using time-dependent positive predictive value (PPV) and negative predictive value (NPV) analysis. We used clinically pertinent PD-L1 expression cut-offs: 0%, 1–49% and ≥50%. One institution reported PD-L1 as 0% vs. ≥1% and we only used PD-L1 = 0% for comparison.

### Statistical analysis

We previously developed the iSEND score by modelling multiple covariates with their optimal cut-offs from the training cohort at the University of Miami.^27^ For the convenience and practicality, the coefficients of the iSEND score were adapted into rounded integers unlike our initial modelling report from the training cohort. In this analysis, the iSEND score was composed as iSEND Score = [‘Male’ = 1 vs. ‘Female’ = 0] + [‘ECOG ≧ 2′ = 1 vs. ‘ECOG < 2′ = 0] + [‘NLR ≧ 5 plus DNLR ≧ 0′ = 2 vs. ‘the others’ = 0]. Patients were categorised into the iSEND Good, Intermediate or Poor groups according to their scores as Good: 0, Intermediate: 1 and Poor:  ≦ 2.

The progression-free survival (PFS) and overall survival (OS) were correlated with each iSEND group in training, independent validation and pooled-analysis groups. Patients with tumour PD-L1 expressions available were selected for performance comparison. The prediction performance of the iSEND Poor group was compared with PD-L1 = 0% group, whereas the iSEND Good group was compared with PD-L1 ≥ 50% group. Time-dependent PPVs and NPVs for the event of overall survival (mortality) at 3, 6, 9 and 12 months were estimated for each pair and obtained p-values. All statistical tests were two-sided, and *p*-values of <0.05 were considered statistically significant.^28^ Analyses involving Cox proportional hazards regression models, log-rank test and logistic regression were performed by using R (http://www.r-project.org) and IBM SPSS (Version 25). Due to heterogeneity of cohorts from different institutions regarding smoking history in different genders, we performed subgroup analysis by using four groups as the following: (1) female with smoking history, (2) male with smoking history, (3) female without smoking history and (4) male without smoking history.

## Results

### General demographics and distribution of clinical and haematologic parameters

**Fig. 1:**
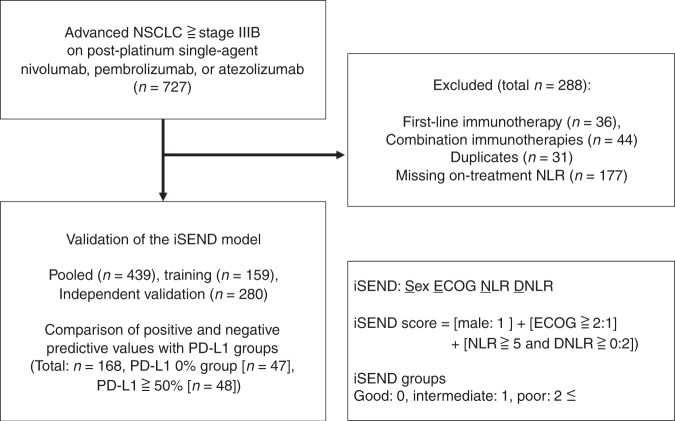
Study flow diagram

We collected a retrospective database of 727 patients with pathologically confirmed diagnosis of NSCLC at advanced stage (higher than or equal to stage IIIB) treated with PD-1/L1 monotherapy after progression on platinum-based treatment (Fig. 1). After excluding patients with first-line PD-1/L1 monotherapy without previous platinum (*n* = 36), treated with concomitant combination treatments including chemoimmunotherapy (*n* = 44), with duplicate entries (*n* = 31), and patients without either baseline NLR or on-treatment NLR (*n* = 177), the analyses were performed in training (*n* = 159), independent validation (*n* = 280) and pooled cohorts (*n* = 439) that met the selection criteria. Treatments included nivolumab (*n* = 327), pembrolizumab (*n* = 64) or atezolizumab (*n* = 48) administered between March 2015 and December 2017 from authors’ institutions: University of Miami (*n* = 159, *n* = 40 for expansion included for the independent validation cohort), Gustave Roussy (*n* = 134), Fukushima University (*n* = 56) and Northwestern University (*n* = 50). In addition, an independent cohort of NSCLC patients (*n* = 68) who received second-line chemotherapy without immunotherapy was collected for comparison from the University of Miami.

Patient demographics including age, sex, smoking history, ECOG-performance status, pathology (squamous vs. non-squamous), the presence of epidermal growth factor receptor (EGFR) or anaplastic lymphoma kinase (ALK) alterations, and the distribution of haematologic variables: baseline neutrophil-to-lymphocyte ratio (NLR) and delta NLR (DNLR = NLR2 [before the second dose] – NLR1 [baseline NLR]) were summarised by pooled (*n* = 439), training (*n* = 159) and independent validation cohorts (*n* = 280) (Table 1). In pooled analysis (*n* = 439), the median patient age was 66 (range: 28–91) and 246 (56%) patients were men. There were 118 squamous cell carcinomas (26.9%) and 321 non-squamous cell carcinomas (73.1%). In total, 357 patients (81.3%) had an ECOG-performance status 0 or 1, whereas 82 patients (18.7%) had ECOG of 2 or above. In total, 352 patients (80.2%) had smoking history and 76 patients (17.3%) had no smoking history. The median of baseline NLR (NLR1) was 4.5 and it ranged from 0.5 to 57 and the median of DNLR was 0.1 and it ranged from −41.7 to 29.7. There was no statistical difference of distribution of the same parameters among training and independent validation cohorts treated with PD-1/L1 monotherapy. In addition, the demographic and haematologic distribution of post-platinum chemotherapy treatment cohort was also analysed. There was no statistically significant distribution between chemotherapy cohorts (*n* = 68) and pooled cohorts on PD-1/L1 monotherapy (*n* = 439) (Supplement 1).

**Table 1:** Demographic and haematologic distribution of pooled, training and independent validation cohorts for PD-1/L1 monotherapy

Variable	Pooled (*n* = 439)		Training (*n* = 159)		Independent validation (*n* = 280)		*p*-value^#^
	*N*	%	*N*	%	*N*	%	
Sex							0.4123
Male	246	56	85	53.5	161	57.5	
Female	193	44	74	46.5	119	42.5	
Smoking history							0.45
Unknown	11	2.5	2	1.3	9	3.2	
Any	352	80.2	132	83	220	78.6	
Never	76	17.3	25	15.7	51	18.2	
ECOG performance							0.2732
ECOG: 0–1	357	81.3	125	78.6	232	82.9	
ECOG: 2–3	82	18.7	34	21.4	48	17.1	
Pathology							0.5397
Squamous	118	26.9	40	25.2	78	27.9	
Non-squamous	321	73.1	119	74.8	202	72.1	
EGFR/ALK alterations							0.9461
Unknown	14	3.2	–	–	14	5	
No	379	86.3	142	89.3	237	84.6	
Yes	46	10.5	17	10.7	29	10.4	
Composite biomarker							0.3547
Others	363	82.7	135	84.9	228	81.4	
NLR ≧ 5 and DNLR ≧ 0	76	17.3	24	15.1	52	18.6	
Median (range)							
Age	66 (28, 91)		68 (41, 90)		66 (28, 91)		0.028
# of prior TX	1 (1, 10)		1 (1, 6)		1 (1, 10)		0.831
NLR1	4.5 (0.5, 57)		4.1 (0.5, 24.1)		4.5 (0.7, 57)		0.17
DNLR	0.1 (−41.7, 29.7)		0.1 (−15.6, 17.1)		0.1 (−41.7, 29.7)		0.631

# Chi-square test for categorical variables and Wilcoxon two-sample test for continuous variables for training and independent validation cohort comparison

*ECOG* Eastern Cooperative Oncology Group, *EGFR* epidermal growth factor receptor, *ALK* anaplastic lymphoma kinase, *NLR* neutrophil-to-lymphocyte ratio, *DNLR* delta NLR, *NLR1* baseline NLR

### Sex, ECOG-performance status, NLR and DNLR

Multivariate analysis was performed to compare covariates of the iSEND model with OS from the pooled, training and independent validation cohorts. Poor performance status with ECOG ≥ 2 continued to show a good correlation with poor OS in pooled (HR, 1.78; 95% CI, 1.36–2.31), training (HR, 2.05; 95% CI, 1.32–3.17) and independent validation (HR, 1.75; 95% CI, 1.25–2.44) cohorts, respectively (Table 2). The composite score (NLR ≥ 5 and DNLR ≥ 0) also maintained a good correlation with poor OS in pooled (HR, 1.81; 95% CI, 1.38–2.38), training (HR, 3.07; 95% CI, 1.89–5.0) and independent validation (HR, 1.47; 95% CI, 1.06–2.05) cohorts, respectively. However, male sex was not associated with worse OS. In the previous modelling report, OS analysis was not available for shorter follow-up at the time of analysis.^27^

**Table 2:** Multivariate analysis for covariates of the iSEND model with OS from pooled, training and independent validation cohorts

	Pooled		Training		Independent validation	
	HR (95% CI)	p-value	HR (95% CI)	*p*-value	HR (95% CI)	*p*-value
Sex: male	1.2 (0.97, 1.49)	0.0941	1.42 (0.98, 2.06)	0.0663	1.02 (0.78, 1.34)	0.8769
ECOG: 2–3	1.78 (1.36, 2.31)	<0.0001	2.05 (1.32, 3.17)	0.0013	1.75 (1.25, 2.44)	0.001
NLR ≥ 5 and DNLR ≥ 0	1.81 (1.38, 2.38)	<0.0001	3.07 (1.89, 5.0)	<0.0001	1.47 (1.06, 2.05)	0.0223

*HR* hazard ratio, *95% CI* 95% confidence interval

### Clinical outcome with different iSEND groups

**Fig. 2:**
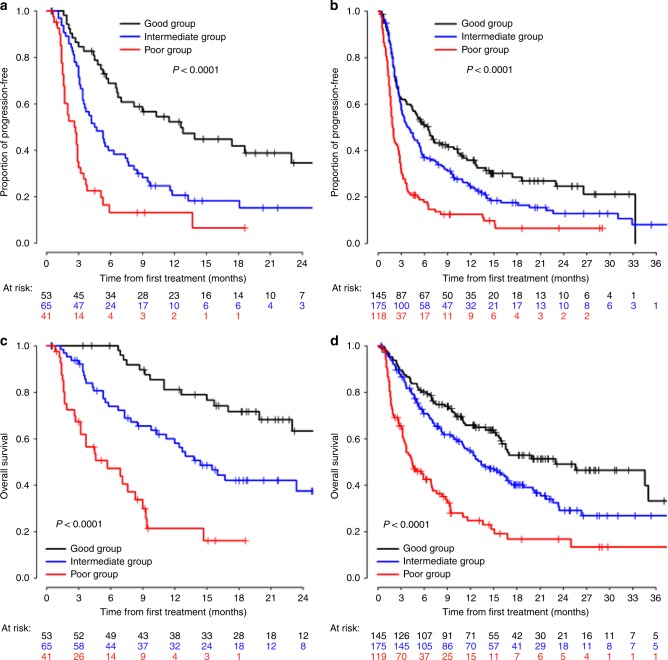
Kaplan–Meier curves of the iSEND groups for PFS and OS in training and pooled cohorts treated with post-platinum PD-1/L1 monotherapy. **a** Training cohort PFS, **b** validation cohort PFS, **c** training OS and **d** validation OS

**Fig. 3:**
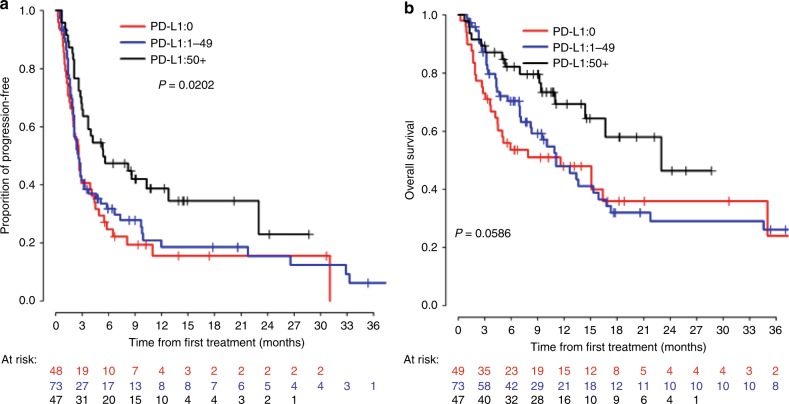
Kaplan–Meier curves for PFS (A) and OS (B) of different PD-L1 groups in PD-1/L1 monotherapy cohort

Median follow-up for pooled-analysis patients (*n* = 439) was 18.4 months (95% CI: 16–20.2) as compared with initial training cohort (*n* = 159), which was 18.9 months (95% CI: 18.3–21.6) by using reverse Kaplan–Meier method (Figs. 2, 3). In total, 145 patients (33%) were grouped into the iSEND Good, 175 patients (40%) into the iSEND Intermediate and 119 patients (27%) into the iSEND Poor as compared with 53, 65 and 41 patients in the training cohort, respectively. The median PFS for patients in the iSEND Good, Intermediate and Poor groups in the pooled cohort were 6.5 (95% CI: 4.4–9.0), 4.0 (95% CI: 3.0–5.3) and 1.9 months (95% CI: 1.6–2.6) as compared with 12.6 (6.7–23), 4.5 (3.4–7.4) and 2.7 (1.7–2.9) in the training cohort, respectively. The median OS for patients in the iSEND Good, Intermediate and Poor groups from the training cohort were 23 (95% CI: 15.9-NR), 13.4 (95% CI: 10.9–17) and 4.5 months (95% CI: 3.4–6.9), respectively as compared with NR, 14.4 (10.3-NE), and 5.7 (3.2–8.3) in the training cohort (*p* < 0.0001). In addition, independent patients (*n* = 68) who received only chemotherapy without immunotherapy were analysed for their PFS for comparison (Supplement 2). The median PFS of the iSEND Good, Intermediate and Poor groups for chemotherapy-treated patients were 9.6 (95% CI: 3.7–15.8), 9.8 (95% CI: 6.7–14.1) and 9.9 months (95% CI: 1.4-NR), respectively (p = 0.9295). This highlights that the iSEND model selectively associates with PD-1/L1 monotherapy in post-platinum setting but not with second-line post-platinum chemotherapy treatment.

### Time-dependent predictive values for mortality and progression among patients categorised by the iSEND model vs. by PD-L1 expression groups

A total of 169 patients had the PD-L1 expressions available and their outcomes were similar to the previous literature outcomes.^3^ Patient characteristics were compared between PD-L1-unavailable patients (*n* = 270) and PD-L1-available patients (*n* = 169) and there was no difference except the distribution of ECOG (Supplement 3). PD-L1-available patients had better ECOG. We compared the prediction performance of the iSEND Poor (*n* = 119) with PD-L1 = 0% (*n* = 47) by time-dependent PPV for mortality (OS) and progression (PFS) (Supplement 4 for mortality, Supplement 5 for progression and Supplement 6 for time-dependent C index). The PPVs for mortality by using the iSEND Poor group were significantly superior compared with PD-L1 = 0% at 12 (0.75 vs. 0.53, p = 0.013), 18 (0.85 vs. 0.65, p = 0.03) and 24 months (0.85 vs. 0.65, p = 0.03), respectively (Table 3). We also compared the prediction performance of the iSEND Good with PD-L1 ≥ 50% by time-dependent NPV for mortality and progression. However, this analysis did not demonstrate any statistical differences.

**Table 3:** Comparison of time-dependent PPV for mortality by the iSEND Poor group vs. PD-L1 0% group

Months	iSEND Poor			PD-L1 0%			*p*-value
	PPV	95% CI		PPV	95% CI		
6	0.57	0.47	0.66	0.46	0.31	0.59	0.18
12	0.75	0.64	0.83	0.53	0.38	0.67	0.01
18	0.85	0.73	0.92	0.65	0.46	0.79	0.03
24	0.85	0.73	0.92	0.65	0.46	0.79	0.03

### The correlation of gender and smoking history with clinical outcomes

Consistent clinical outcome association with covariates of the iSEND model was demonstrated, including ECOG and composite biomarkers (NLR and DNLR) between training and independent validation cohorts, but the clinical outcome association with sex showed variability. The male association with poor PFS in multivariate analysis from the training cohort (HR = 1.92, 95% CI: 1.26–2.94, *p* = 0.003) was neither consistent with OS from the training cohort nor with PFS or OS from the independent validation cohort. In an independent multivariate analysis including ECOG, composite biomarker and smoking history for the patients in the pooled cohort, patients with no smoking history were strongly associated with poor PFS (HR, 1.96; 95% CI, 1.47–2.61) as well as OS (HR, 1.58; 95% CI, 1.14–2.20) but to a less degree (Supplement 7). However, no statistical correlation was observed between poor OS and other factors like comorbidities, age older than the median of 65 or above second-line prior treatment.

Consequently, heterogeneous trends in the proportion of patients with smoking history in different sexes were observed, and we regrouped the patients into four groups as the following: (1) female with smoking history, (2) male with smoking history, 3) female without smoking history and 4) male without smoking history (Supplement 8). Each group had significantly different clinical outcomes in PFS and OS (Supplement 9). The median PFS for each group was 5.5 (3.8–7.2), 3.2 (2.8–4.1), 2.1 (1.6–3.7) and 1.9 (1.4–2.8) months, respectively (log-rank, *p* < 0.001). The median OS was 15.7 (12.6–25), 12.4 (9.4–16), 7 (5.1–21.1) and 7.1 (4.4–11.6) months, respectively (log-rank, *p* < 0.028).

## Discussion

This article demonstrates an independent validation and pooled analysis of the iSEND model in advanced NSCLC patients treated with PD-1/L1 monotherapy after progression on platinum in international cohorts. The iSEND model was previously developed to identify the resistant patients with high baseline NLR and on-treatment increase of NLR during PD-1/L1 monotherapy. The highest coefficient designed for composite biomarkers of NLR and positive DNLR from the training cohort maintained to have a high hazard ratio in our multivariate analysis in both independent validation and pooled cohorts. The iSEND Poor group correlated consistently with poor outcome and had superior prediction performance compared with the PD-L1 = 0% group. However, the iSEND Good group did not have a significantly different performance compared with the PD-L1 ≥ 50% group. Neither the iSEND model nor PD-L1 high expression had better association with significantly superior clinical outcomes. In summary, the iSEND Poor group has a better correlation with poor outcome of PD-1/L1 monotherapy than PD-L1 = 0% in post-platinum setting.

In our pooled cohort, there were only 169 patients who had tumour PD-L1 expression available from 439 patients because most patients included in the study were collected when nivolumab was initially FDA-approved in post-platinum setting without requirement of PD-L1 expression. As a result, iSEND score could be derived from everyone regardless of PD-L1 expression availability. Importantly, the iSEND model showed a specific association with PD-1/L1 monotherapy outcome but not with an independent second-line post-platinum chemotherapy cohort, suggesting potential predictive value if tested prospectively. Overall, the iSEND Poor group would not benefit from PD-1/L1 monotherapy and it suggests that this population may benefit from different treatment options. Practically, considering the rapidly reshaping landscape of lung cancer immunotherapy with new FDA approvals, most patients would have used PD-1/L1 chemoimmunotherapy as first line in advanced setting. Evaluation of the iSEND model in this first-line chemoimmunotherapy population is warranted. This model may be of value if validated in an adjuvant setting, where iSEND Poor may not benefit from PD-1/L1 monotherapy.

Furthermore, in multivariate analysis of the independent validation cohort, female sex showed no association with favoured clinical outcome unlike in the training cohort. Male gender was a significant risk factor in initial training cohort using the data lock of March 2017 but when the data lock date was extended for 9 more months for December 2017 for validation work, many patients who were initially censored have had events in validation dataset, which may explain the discrepancy of insignificant hazard ratio of male gender in training cohort in repeat evaluation included in this study. Women are generally known to have better clinical outcome in NSCLC patients from previous literatures; however, in a recent large-scale pan-cancer meta-analysis, the opposite finding was reported.^17,27,29,30^ Importantly, in this pooled meta-analysis report, different disease ontologies treated with PD-1/L1 monotherapy were pooled together, and the two among six published clinical trials selected for NSCLC did not favour outcome for male patients. Smoking tobacco is highly carcinogenic and has an important and interesting implication in lung cancer patients on immunotherapy for the mutagenic potential, which may lead to higher tumour mutational burden (TMB). In addition to the primary objective of this study to validate the training model, we explored the potential aetiology of discrepant correlations of sex to clinical outcomes across different cohorts. We regrouped the patients by different sexes and any smoking history for additional analysis. The clinical outcome for females with smoking history showed significantly better outcomes in their PFS and OS, which suggests that smoking history is more important and further modelling should consider this parameter.^31^ Smoking history may correlate with higher TMB, but it may worsen the outcome associated with other comorbidities, therefore affecting more on PFS than on OS. In our pooled cohort, females with smoking history had the best PFS and OS. Males with smoking history did better than females without smoking history in PFS, but OS appears to be similar in both after about 15 months. Smoking history showed a significant trend but was not added to the model as the primary objective was to validate and compare the performance of the model.

Protumoural role of neutrophils in chronic inflammation, carcinogenesis and metastasis has been well-reported in the animal models in the literature.^32,33^ One of the extrinsic resistance mechanisms to immunotherapy can be proposed arguably by the dominance of high tumour-associated neutrophils (TAN) over cytotoxic lymphocytes in peripheral circulation.^34,35^ We hypothesised that the iSEND Poor group may identify this extrinsically resistant phenotype to immunotherapy. Neutrophil is a type of myeloid cells recruited first at frontline for host immune defence, but literatures have described this effort being affected by different factors such as tumour growth factor-β (TGF-β), interleukin 1, 6, 10, arginase and chemokine (C–X–C motif) ligand 15 (CXCL 15), which in turn, polarise TAN to contribute for immunosuppressive tumour microenvironment.^36,37^ The mechanism is poorly understood and discovering this mechanism will be of great importance, however, it is beyond the scope of this clinical finding. Yet, the immunosuppressive role of neutrophils is reported in different cancers, and high PD-L1-expressed tumour as well as neutrophils are suggested to suppress cytotoxic T cells.

The landscape of immunotherapy for advanced NSCLC is continuously reshaping. Despite this triumphant advance in immunotherapy, resistance is common, and patients unfortunately succumb to their disease. Deciphering resistance mechanisms and discovering the novel agents to overcome such resistances can significantly improve the patient outcome. This independent validation and exploratory analysis from multicentre international cohorts reproduced identification of the resistant patient group by the iSEND Poor. This model was built by using simple clinical and analytical variables to predict outcomes of currently most frequently used immunotherapy, the PD-1/L1 inhibitors. In a true personalised immunotherapy, more important covariates will be required such as different genomic markers and are under active investigations. However, a simple and readily available model can have a strength when its correlation is good and reproducible. This limited finding is from retrospective analysis and rigorous prospective validation is encouraged.

## Supplementary information

Legends, all the supplemental, tables, figures,

## Data Availability

All the data supporting our findings are included within the paper. The datasets used and/or analysed during this study are available from the corresponding author on reasonable request.
