# Supplementary material for: Association of the prognostic model iSEND with PD-1/L1 monotherapy outcome in non-small-cell lung cancer
Source: Br J Cancer. 2019 Nov 25;122(3):340–7. doi: 10.1038/s41416-019-0643-y (PMC7000664; doi:10.1038/s41416-019-0643-y)
Supplement: Supplementary file 1 — Legends, all the supplemental, tables, figures, [file 41416_2019_643_MOESM1_ESM.pptx]

## Slide 1
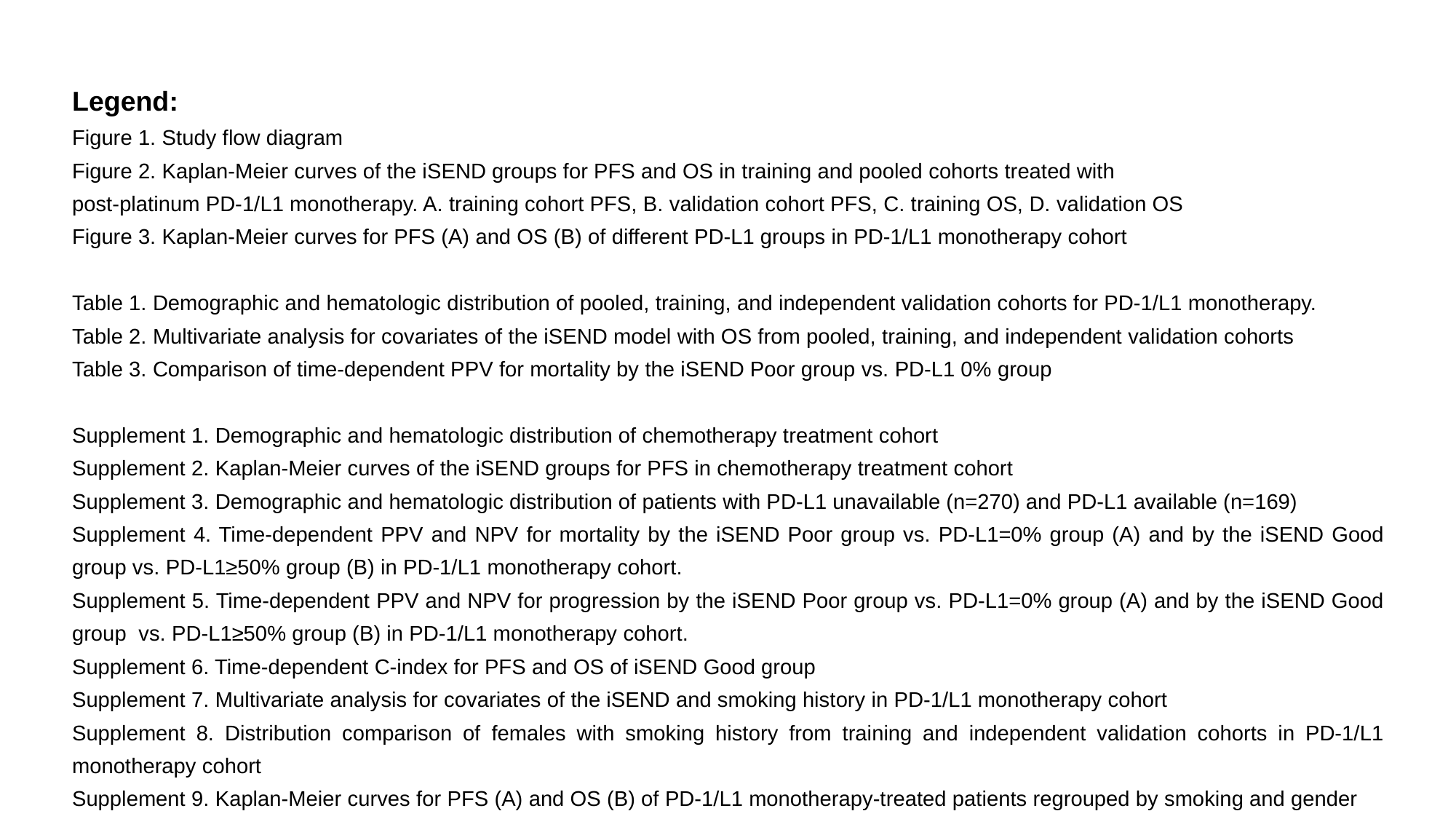

Legend:
Figure 1. Study flow diagram
Figure 2. Kaplan-Meier curves of the iSEND groups for PFS and OS in training and pooled cohorts treated with
post-platinum PD-1/L1 monotherapy. A. training cohort PFS, B. validation cohort PFS, C. training OS, D. validation OS
Figure 3. Kaplan-Meier curves for PFS (A) and OS (B) of different PD-L1 groups in PD-1/L1 monotherapy cohort
Table 1. Demographic and hematologic distribution of pooled, training, and independent validation cohorts for PD-1/L1 monotherapy.
Table 2. Multivariate analysis for covariates of the iSEND model with OS from pooled, training, and independent validation cohorts
Table 3. Comparison of time-dependent PPV for mortality by the iSEND Poor group vs. PD-L1 0% group
Supplement 1. Demographic and hematologic distribution of chemotherapy treatment cohort
Supplement 2. Kaplan-Meier curves of the iSEND groups for PFS in chemotherapy treatment cohort
Supplement 3. Demographic and hematologic distribution of patients with PD-L1 unavailable (n=270) and PD-L1 available (n=169)
Supplement 4. Time-dependent PPV and NPV for mortality by the iSEND Poor group vs. PD-L1=0% group (A) and by the iSEND Good group vs. PD-L1≥50% group (B) in PD-1/L1 monotherapy cohort.
Supplement 5. Time-dependent PPV and NPV for progression by the iSEND Poor group vs. PD-L1=0% group (A) and by the iSEND Good group vs. PD-L1≥50% group (B) in PD-1/L1 monotherapy cohort.
Supplement 6. Time-dependent C-index for PFS and OS of iSEND Good group
Supplement 7. Multivariate analysis for covariates of the iSEND and smoking history in PD-1/L1 monotherapy cohort
Supplement 8. Distribution comparison of females with smoking history from training and independent validation cohorts in PD-1/L1 monotherapy cohort
Supplement 9. Kaplan-Meier curves for PFS (A) and OS (B) of PD-1/L1 monotherapy-treated patients regrouped by smoking and gender

## Slide 2
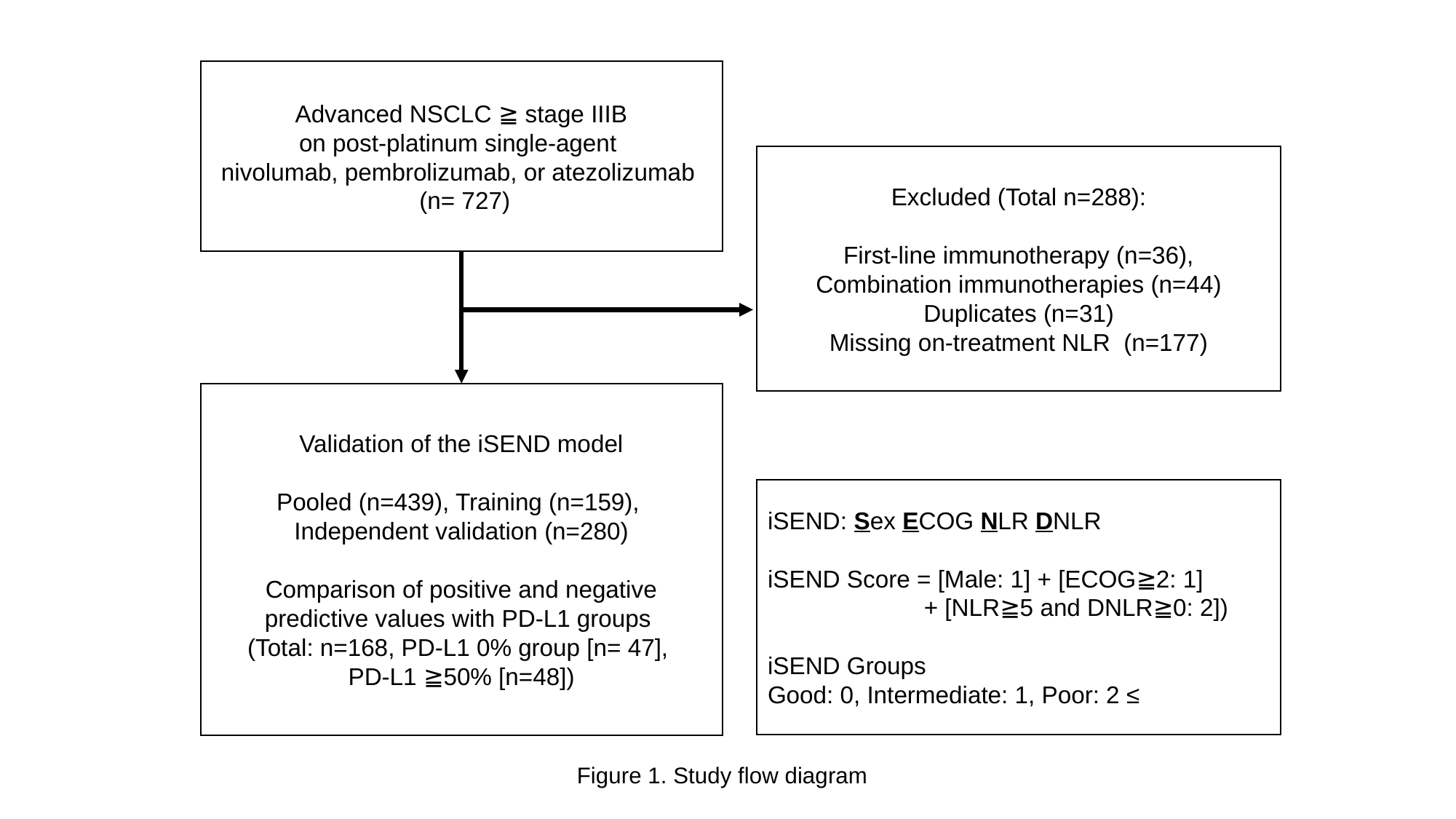

Advanced NSCLC ≧ stage IIIB
on post-platinum single-agent
nivolumab, pembrolizumab, or atezolizumab
 (n= 727)
Excluded (Total n=288):
First-line immunotherapy (n=36),
Combination immunotherapies (n=44)
Duplicates (n=31)
Missing on-treatment NLR (n=177)
Validation of the iSEND model
Pooled (n=439), Training (n=159),
Independent validation (n=280)
Comparison of positive and negative predictive values with PD-L1 groups
(Total: n=168, PD-L1 0% group [n= 47],
PD-L1 ≧50% [n=48])
iSEND: Sex ECOG NLR DNLR
iSEND Score = [Male: 1] + [ECOG≧2: 1]
	 + [NLR≧5 and DNLR≧0: 2])
iSEND Groups
Good: 0, Intermediate: 1, Poor: 2 ≤
Figure 1. Study flow diagram

## Slide 3
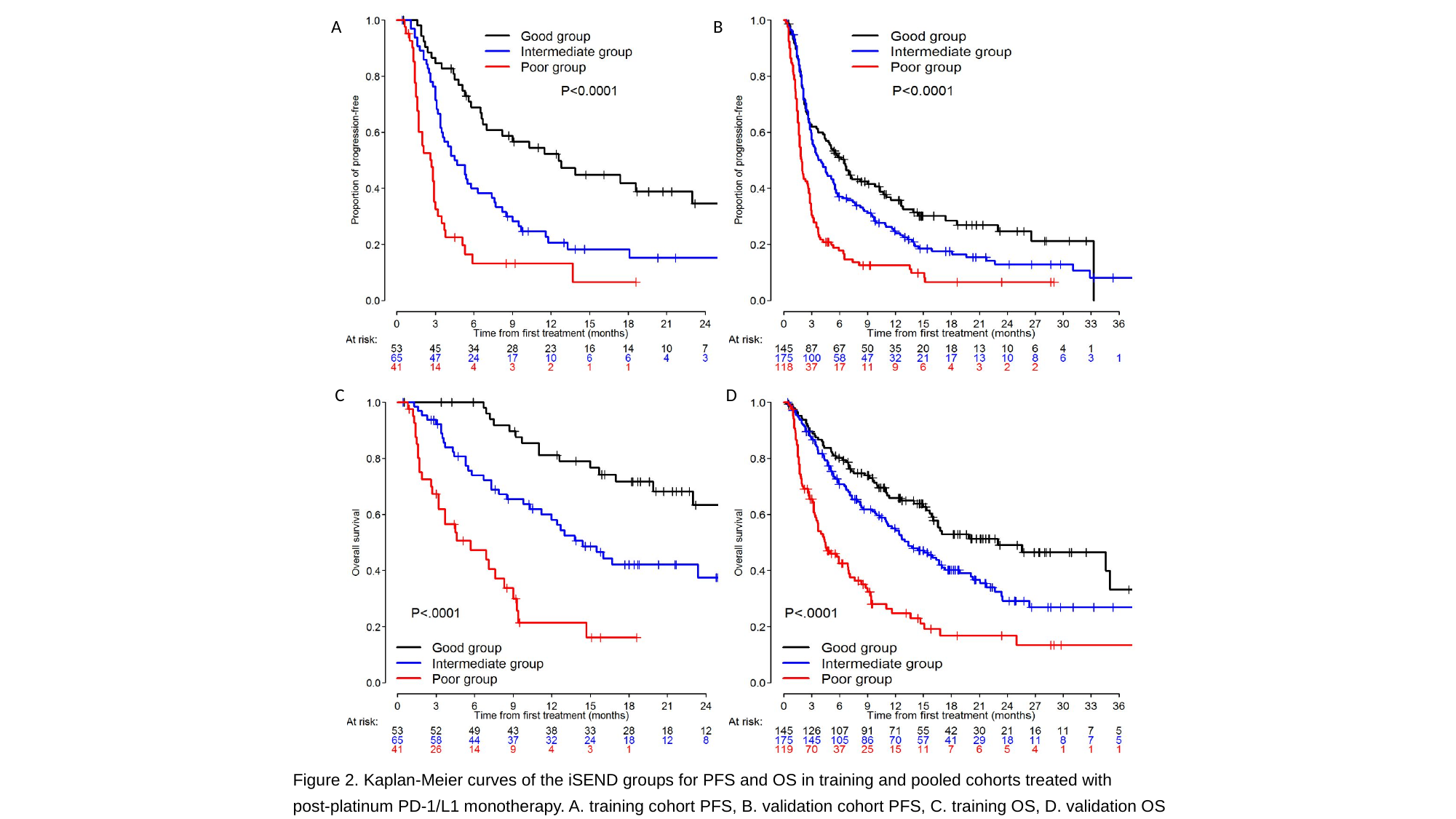

B
A
C
D
Figure 2. Kaplan-Meier curves of the iSEND groups for PFS and OS in training and pooled cohorts treated with
post-platinum PD-1/L1 monotherapy. A. training cohort PFS, B. validation cohort PFS, C. training OS, D. validation OS

## Slide 4
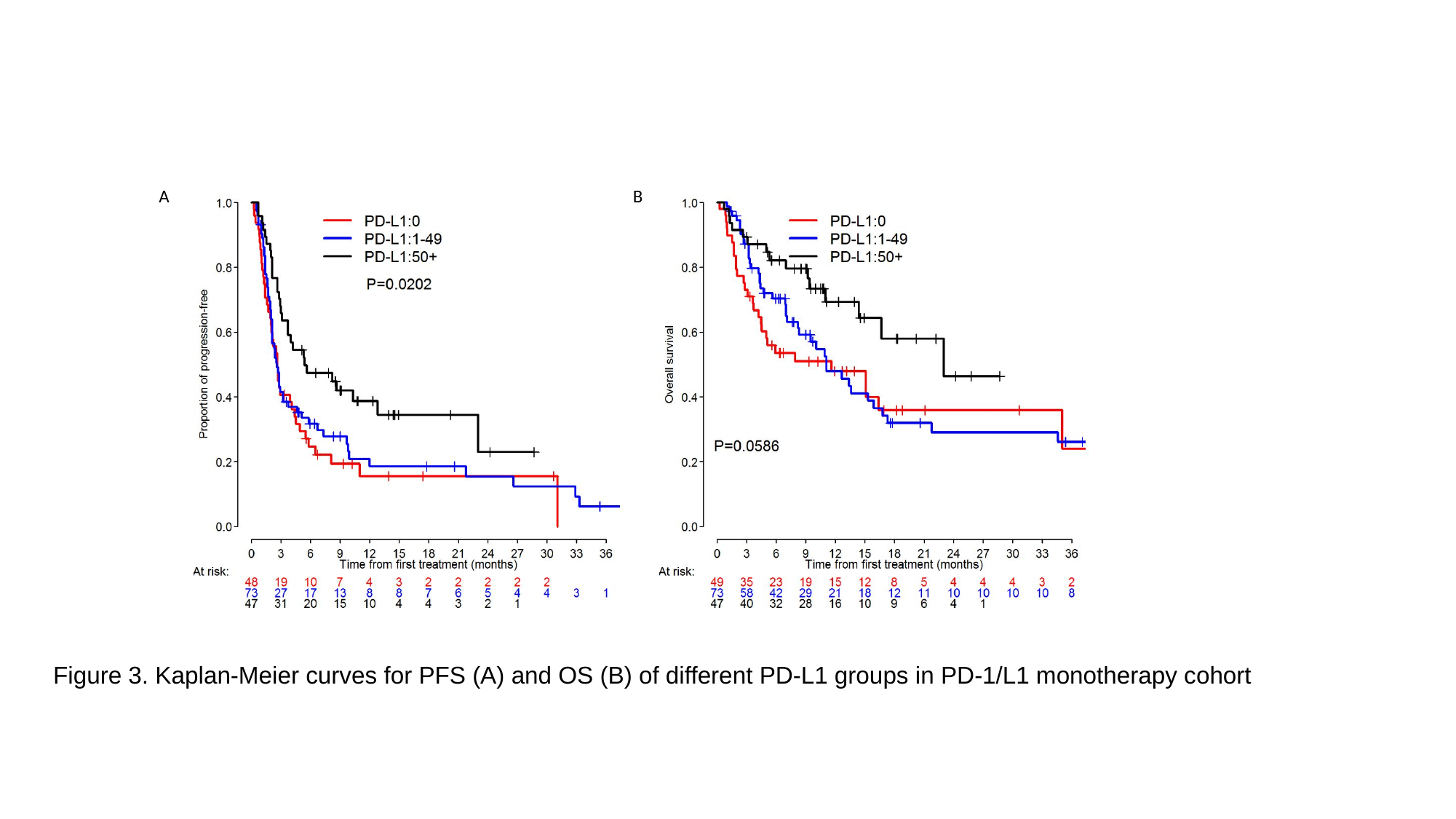

A
B
Figure 3. Kaplan-Meier curves for PFS (A) and OS (B) of different PD-L1 groups in PD-1/L1 monotherapy cohort

## Slide 5
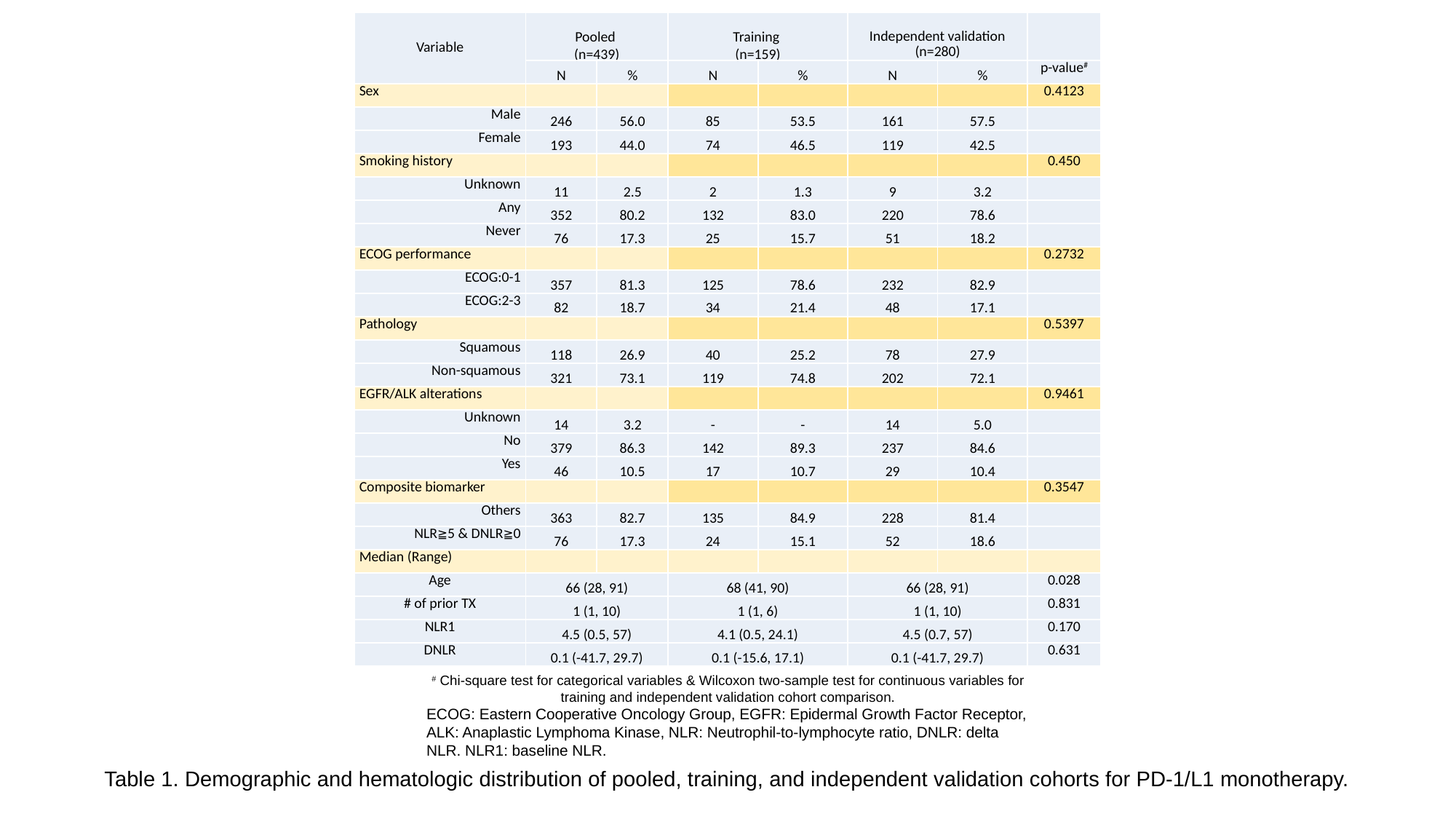

| Variable | Pooled (n=439) | | Training (n=159) | | Independent validation (n=280) | | |
| --- | --- | --- | --- | --- | --- | --- | --- |
| | N | % | N | % | N | % | p-value# |
| Sex | | | | | | | 0.4123 |
| Male | 246 | 56.0 | 85 | 53.5 | 161 | 57.5 | |
| Female | 193 | 44.0 | 74 | 46.5 | 119 | 42.5 | |
| Smoking history | | | | | | | 0.450 |
| Unknown | 11 | 2.5 | 2 | 1.3 | 9 | 3.2 | |
| Any | 352 | 80.2 | 132 | 83.0 | 220 | 78.6 | |
| Never | 76 | 17.3 | 25 | 15.7 | 51 | 18.2 | |
| ECOG performance | | | | | | | 0.2732 |
| ECOG:0-1 | 357 | 81.3 | 125 | 78.6 | 232 | 82.9 | |
| ECOG:2-3 | 82 | 18.7 | 34 | 21.4 | 48 | 17.1 | |
| Pathology | | | | | | | 0.5397 |
| Squamous | 118 | 26.9 | 40 | 25.2 | 78 | 27.9 | |
| Non-squamous | 321 | 73.1 | 119 | 74.8 | 202 | 72.1 | |
| EGFR/ALK alterations | | | | | | | 0.9461 |
| Unknown | 14 | 3.2 | - | - | 14 | 5.0 | |
| No | 379 | 86.3 | 142 | 89.3 | 237 | 84.6 | |
| Yes | 46 | 10.5 | 17 | 10.7 | 29 | 10.4 | |
| Composite biomarker | | | | | | | 0.3547 |
| Others | 363 | 82.7 | 135 | 84.9 | 228 | 81.4 | |
| NLR≧5 & DNLR≧0 | 76 | 17.3 | 24 | 15.1 | 52 | 18.6 | |
| Median (Range) | | | | | | | |
| Age | 66 (28, 91) | | 68 (41, 90) | | 66 (28, 91) | | 0.028 |
| # of prior TX | 1 (1, 10) | | 1 (1, 6) | | 1 (1, 10) | | 0.831 |
| NLR1 | 4.5 (0.5, 57) | | 4.1 (0.5, 24.1) | | 4.5 (0.7, 57) | | 0.170 |
| DNLR | 0.1 (-41.7, 29.7) | | 0.1 (-15.6, 17.1) | | 0.1 (-41.7, 29.7) | | 0.631 |
# Chi-square test for categorical variables & Wilcoxon two-sample test for continuous variables for training and independent validation cohort comparison.
ECOG: Eastern Cooperative Oncology Group, EGFR: Epidermal Growth Factor Receptor, ALK: Anaplastic Lymphoma Kinase, NLR: Neutrophil-to-lymphocyte ratio, DNLR: delta NLR. NLR1: baseline NLR.
Table 1. Demographic and hematologic distribution of pooled, training, and independent validation cohorts for PD-1/L1 monotherapy.

## Slide 6
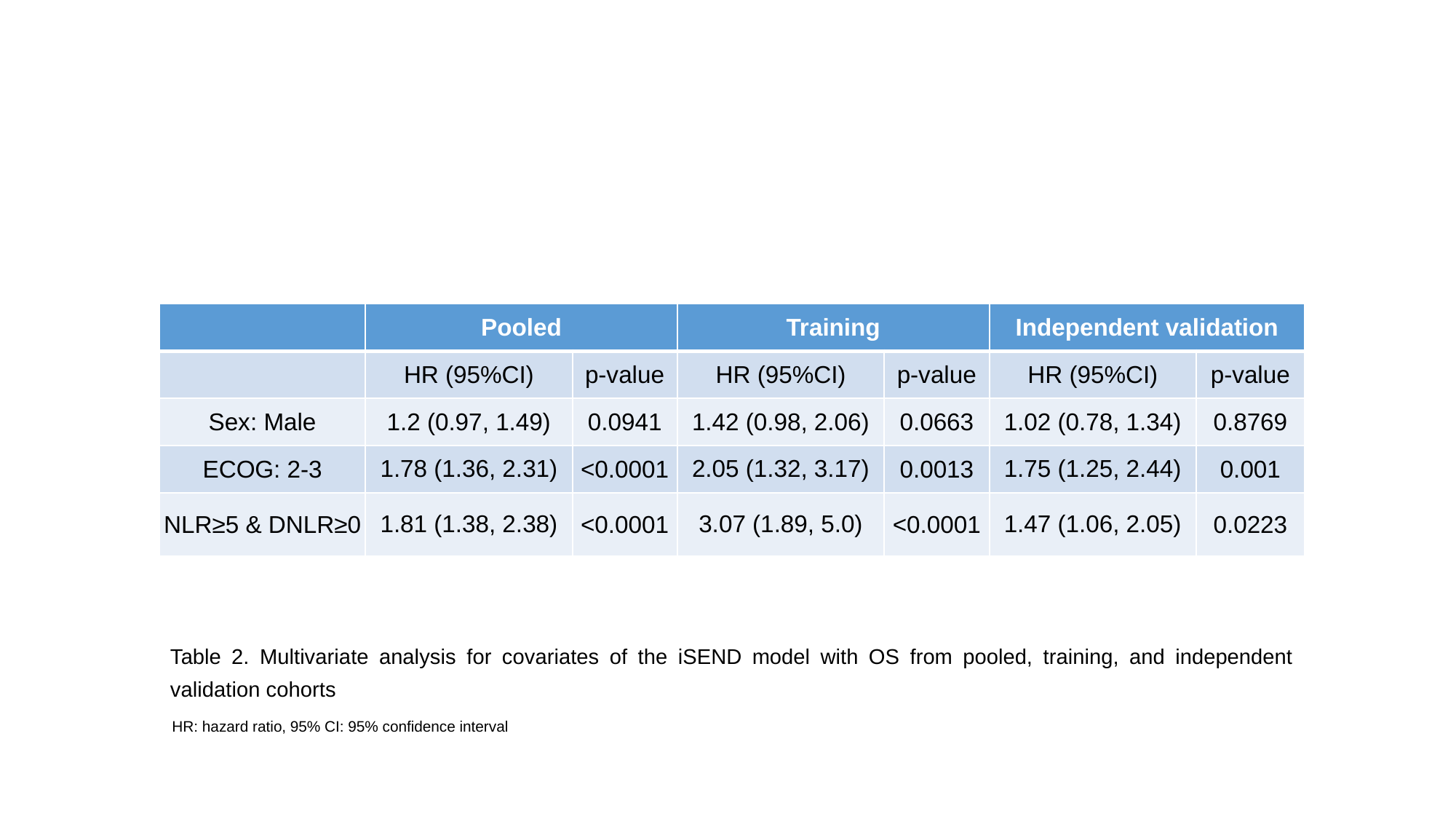

| | Pooled | | Training | | Independent validation | |
| --- | --- | --- | --- | --- | --- | --- |
| | HR (95%CI) | p-value | HR (95%CI) | p-value | HR (95%CI) | p-value |
| Sex: Male | 1.2 (0.97, 1.49) | 0.0941 | 1.42 (0.98, 2.06) | 0.0663 | 1.02 (0.78, 1.34) | 0.8769 |
| ECOG: 2-3 | 1.78 (1.36, 2.31) | <0.0001 | 2.05 (1.32, 3.17) | 0.0013 | 1.75 (1.25, 2.44) | 0.001 |
| NLR≥5 & DNLR≥0 | 1.81 (1.38, 2.38) | <0.0001 | 3.07 (1.89, 5.0) | <0.0001 | 1.47 (1.06, 2.05) | 0.0223 |
Table 2. Multivariate analysis for covariates of the iSEND model with OS from pooled, training, and independent validation cohorts
HR: hazard ratio, 95% CI: 95% confidence interval

## Slide 7
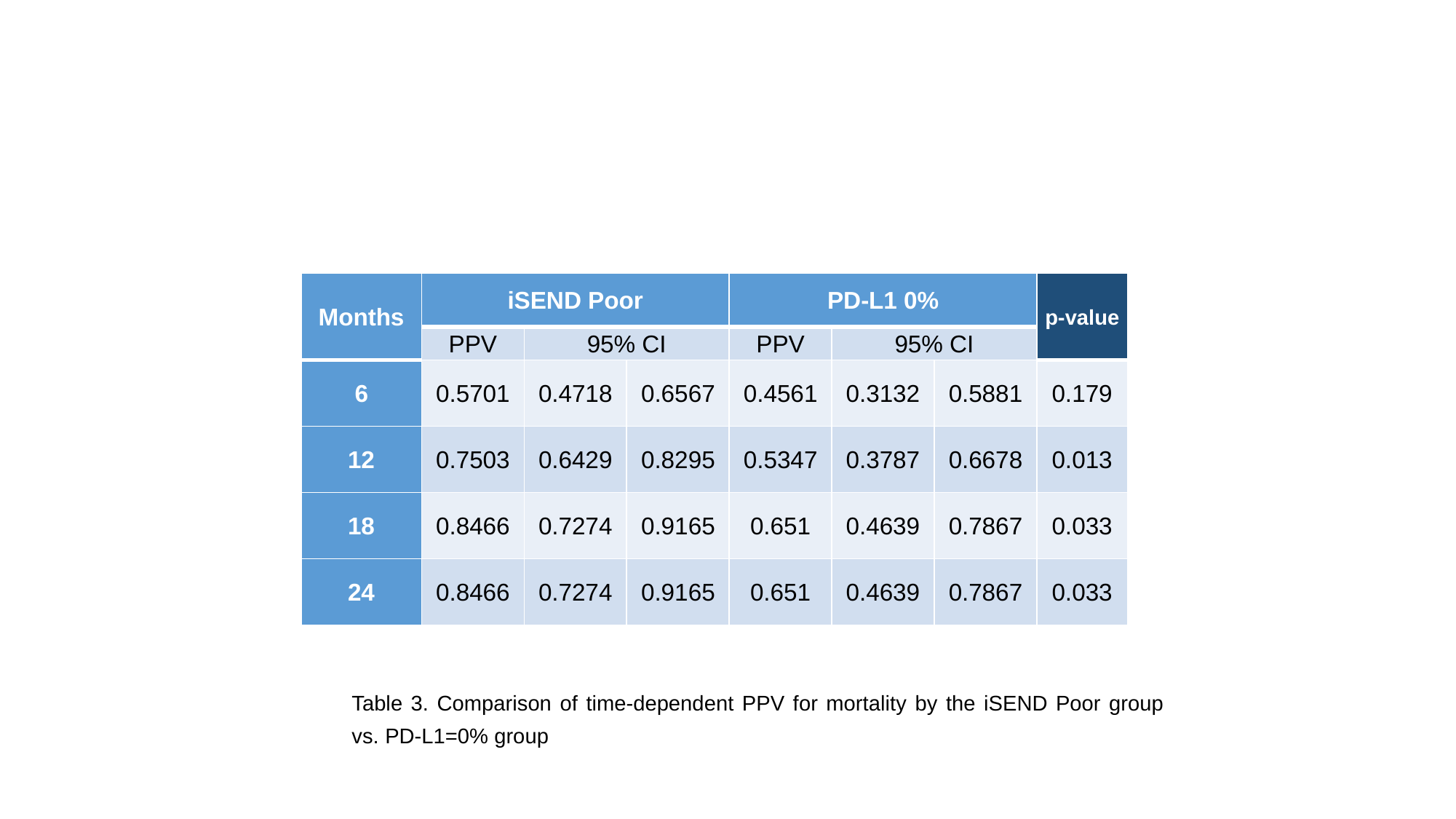

| Months | iSEND Poor | | | PD-L1 0% | | | p-value |
| --- | --- | --- | --- | --- | --- | --- | --- |
| | PPV | 95% CI | | PPV | 95% CI | | |
| 6 | 0.5701 | 0.4718 | 0.6567 | 0.4561 | 0.3132 | 0.5881 | 0.179 |
| 12 | 0.7503 | 0.6429 | 0.8295 | 0.5347 | 0.3787 | 0.6678 | 0.013 |
| 18 | 0.8466 | 0.7274 | 0.9165 | 0.651 | 0.4639 | 0.7867 | 0.033 |
| 24 | 0.8466 | 0.7274 | 0.9165 | 0.651 | 0.4639 | 0.7867 | 0.033 |
Table 3. Comparison of time-dependent PPV for mortality by the iSEND Poor group vs. PD-L1=0% group

## Slide 8
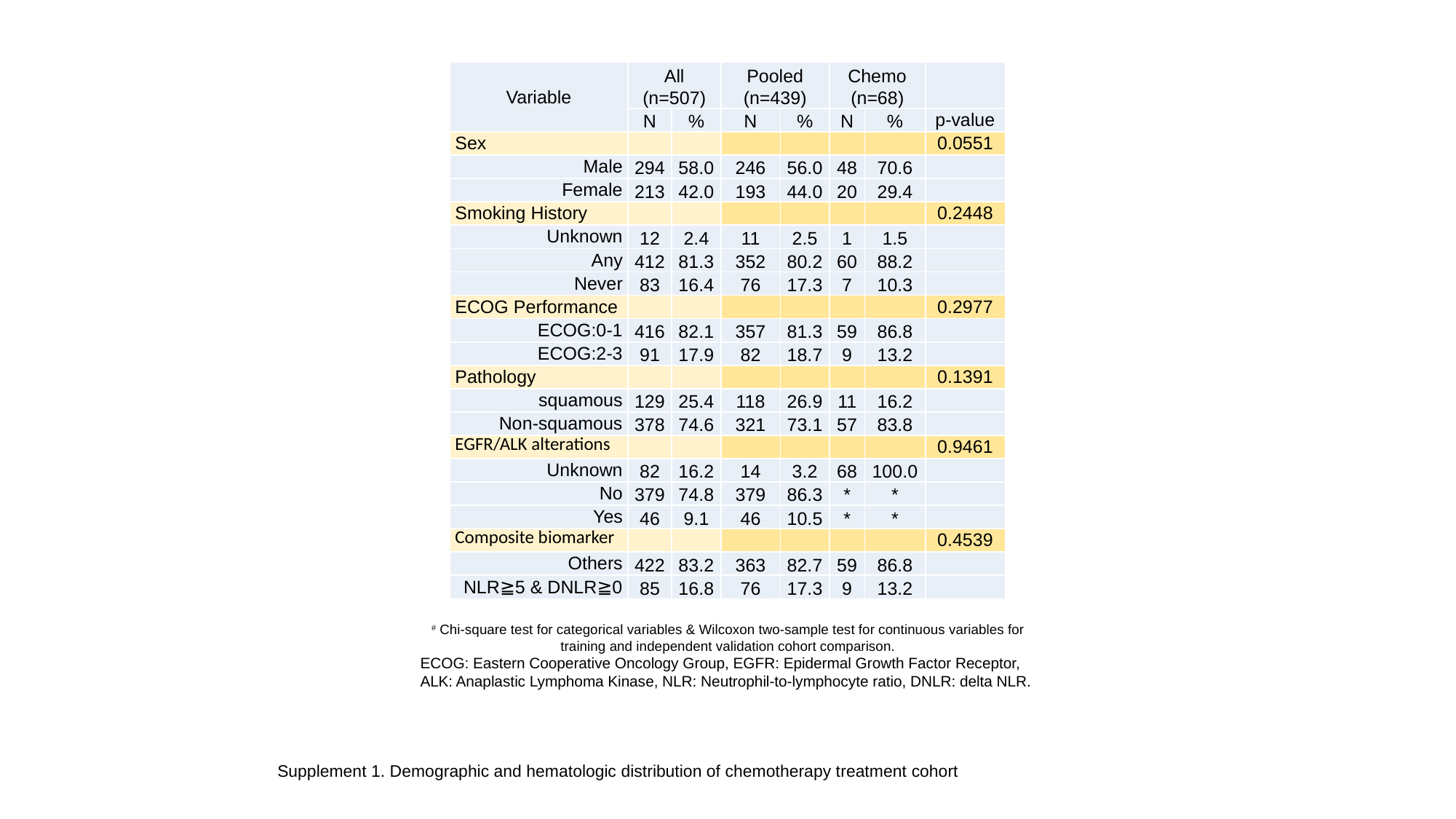

| Variable | All (n=507) | | Pooled (n=439) | | Chemo (n=68) | | |
| --- | --- | --- | --- | --- | --- | --- | --- |
| | N | % | N | % | N | % | p-value |
| Sex | | | | | | | 0.0551 |
| Male | 294 | 58.0 | 246 | 56.0 | 48 | 70.6 | |
| Female | 213 | 42.0 | 193 | 44.0 | 20 | 29.4 | |
| Smoking History | | | | | | | 0.2448 |
| Unknown | 12 | 2.4 | 11 | 2.5 | 1 | 1.5 | |
| Any | 412 | 81.3 | 352 | 80.2 | 60 | 88.2 | |
| Never | 83 | 16.4 | 76 | 17.3 | 7 | 10.3 | |
| ECOG Performance | | | | | | | 0.2977 |
| ECOG:0-1 | 416 | 82.1 | 357 | 81.3 | 59 | 86.8 | |
| ECOG:2-3 | 91 | 17.9 | 82 | 18.7 | 9 | 13.2 | |
| Pathology | | | | | | | 0.1391 |
| squamous | 129 | 25.4 | 118 | 26.9 | 11 | 16.2 | |
| Non-squamous | 378 | 74.6 | 321 | 73.1 | 57 | 83.8 | |
| EGFR/ALK alterations | | | | | | | 0.9461 |
| Unknown | 82 | 16.2 | 14 | 3.2 | 68 | 100.0 | |
| No | 379 | 74.8 | 379 | 86.3 | \* | \* | |
| Yes | 46 | 9.1 | 46 | 10.5 | \* | \* | |
| Composite biomarker | | | | | | | 0.4539 |
| Others | 422 | 83.2 | 363 | 82.7 | 59 | 86.8 | |
| NLR≧5 & DNLR≧0 | 85 | 16.8 | 76 | 17.3 | 9 | 13.2 | |
# Chi-square test for categorical variables & Wilcoxon two-sample test for continuous variables for training and independent validation cohort comparison.
ECOG: Eastern Cooperative Oncology Group, EGFR: Epidermal Growth Factor Receptor, ALK: Anaplastic Lymphoma Kinase, NLR: Neutrophil-to-lymphocyte ratio, DNLR: delta NLR.
Supplement 1. Demographic and hematologic distribution of chemotherapy treatment cohort

## Slide 9
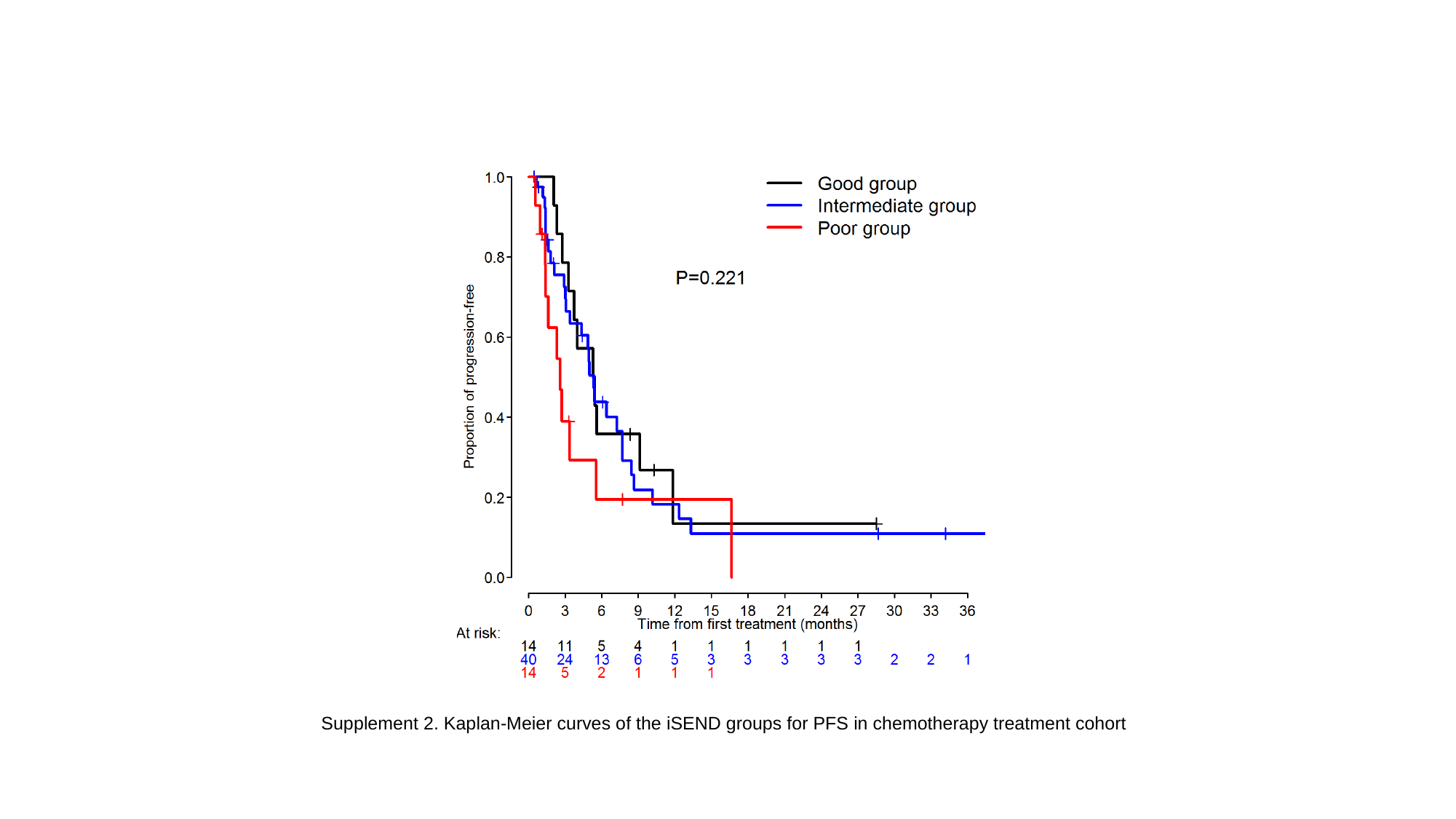

Supplement 2. Kaplan-Meier curves of the iSEND groups for PFS in chemotherapy treatment cohort

## Slide 10
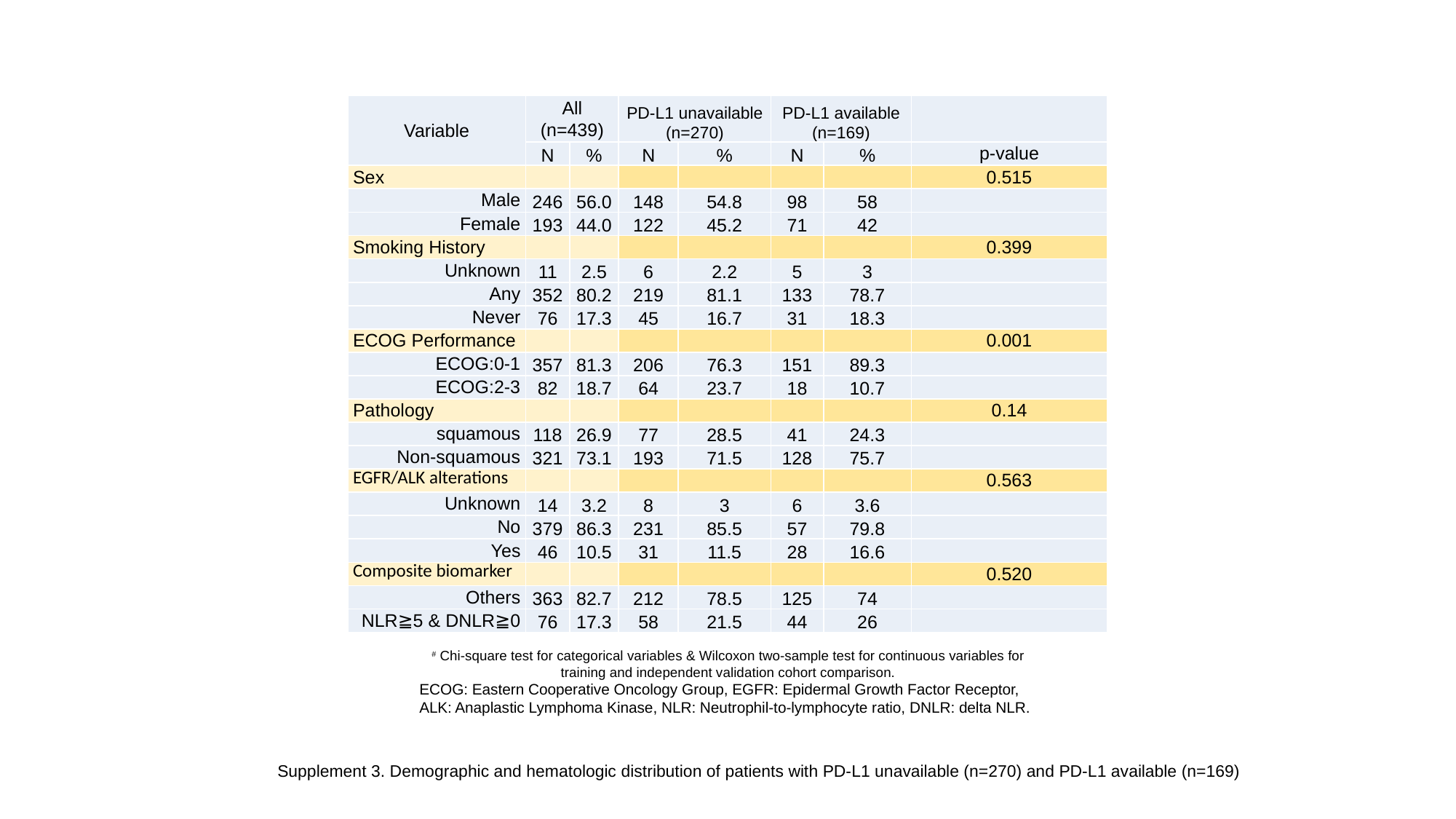

| Variable | All (n=439) | | PD-L1 unavailable (n=270) | | PD-L1 available (n=169) | | |
| --- | --- | --- | --- | --- | --- | --- | --- |
| | N | % | N | % | N | % | p-value |
| Sex | | | | | | | 0.515 |
| Male | 246 | 56.0 | 148 | 54.8 | 98 | 58 | |
| Female | 193 | 44.0 | 122 | 45.2 | 71 | 42 | |
| Smoking History | | | | | | | 0.399 |
| Unknown | 11 | 2.5 | 6 | 2.2 | 5 | 3 | |
| Any | 352 | 80.2 | 219 | 81.1 | 133 | 78.7 | |
| Never | 76 | 17.3 | 45 | 16.7 | 31 | 18.3 | |
| ECOG Performance | | | | | | | 0.001 |
| ECOG:0-1 | 357 | 81.3 | 206 | 76.3 | 151 | 89.3 | |
| ECOG:2-3 | 82 | 18.7 | 64 | 23.7 | 18 | 10.7 | |
| Pathology | | | | | | | 0.14 |
| squamous | 118 | 26.9 | 77 | 28.5 | 41 | 24.3 | |
| Non-squamous | 321 | 73.1 | 193 | 71.5 | 128 | 75.7 | |
| EGFR/ALK alterations | | | | | | | 0.563 |
| Unknown | 14 | 3.2 | 8 | 3 | 6 | 3.6 | |
| No | 379 | 86.3 | 231 | 85.5 | 57 | 79.8 | |
| Yes | 46 | 10.5 | 31 | 11.5 | 28 | 16.6 | |
| Composite biomarker | | | | | | | 0.520 |
| Others | 363 | 82.7 | 212 | 78.5 | 125 | 74 | |
| NLR≧5 & DNLR≧0 | 76 | 17.3 | 58 | 21.5 | 44 | 26 | |
# Chi-square test for categorical variables & Wilcoxon two-sample test for continuous variables for training and independent validation cohort comparison.
ECOG: Eastern Cooperative Oncology Group, EGFR: Epidermal Growth Factor Receptor, ALK: Anaplastic Lymphoma Kinase, NLR: Neutrophil-to-lymphocyte ratio, DNLR: delta NLR.
Supplement 3. Demographic and hematologic distribution of patients with PD-L1 unavailable (n=270) and PD-L1 available (n=169)

## Slide 11
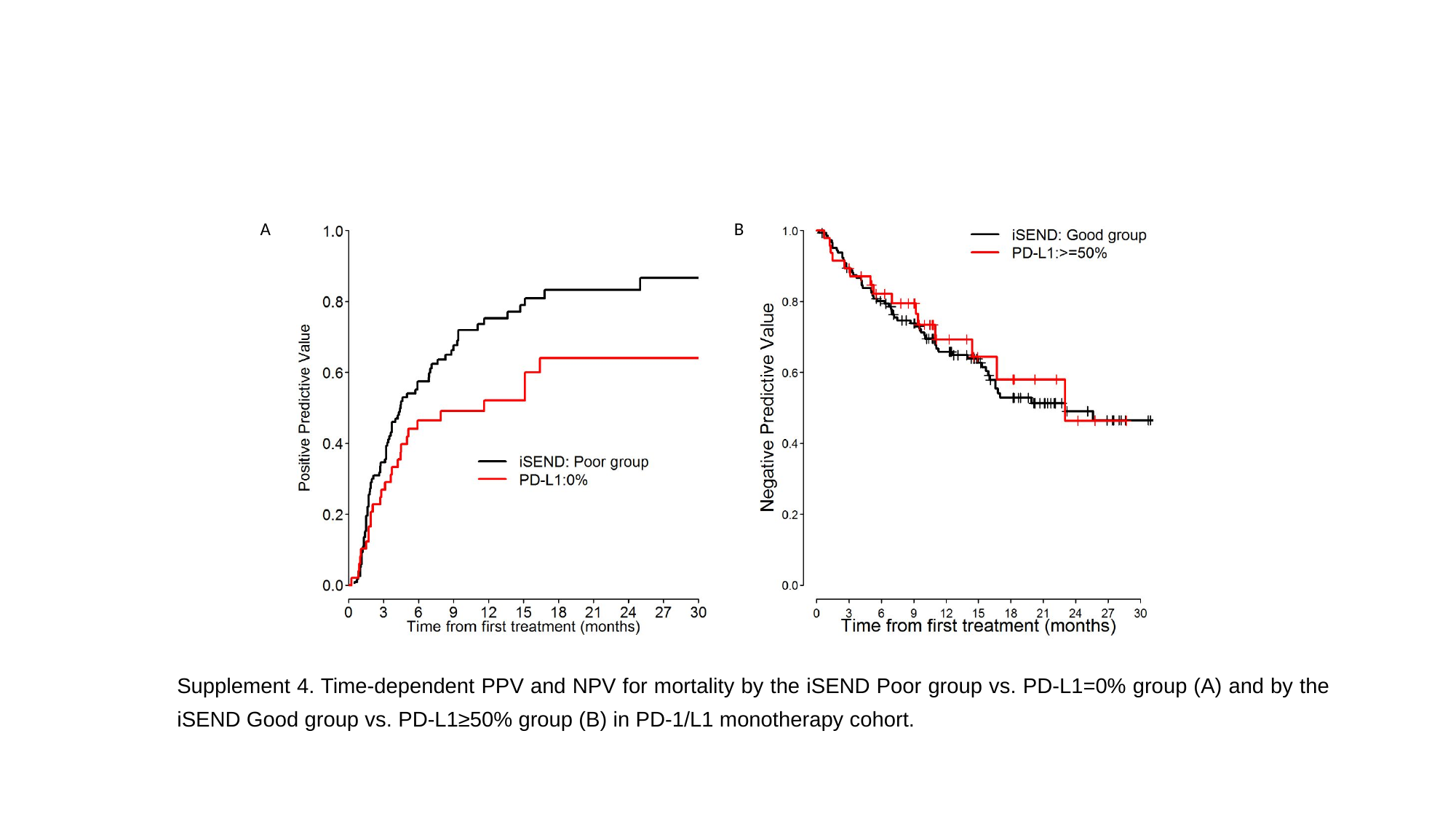

A
B
Supplement 4. Time-dependent PPV and NPV for mortality by the iSEND Poor group vs. PD-L1=0% group (A) and by the iSEND Good group vs. PD-L1≥50% group (B) in PD-1/L1 monotherapy cohort.

## Slide 12
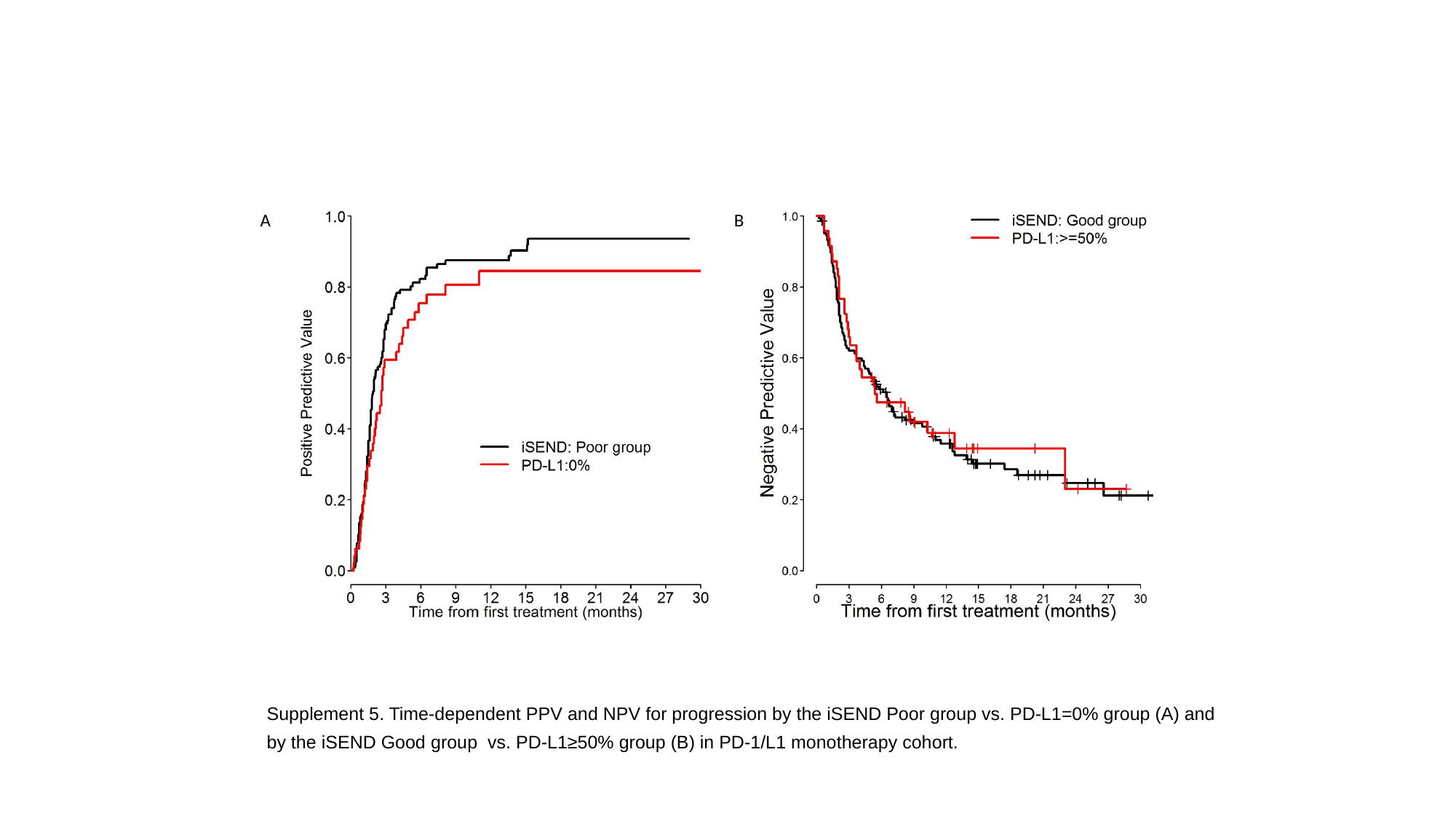

A
B
Supplement 5. Time-dependent PPV and NPV for progression by the iSEND Poor group vs. PD-L1=0% group (A) and
by the iSEND Good group vs. PD-L1≥50% group (B) in PD-1/L1 monotherapy cohort.

## Slide 13
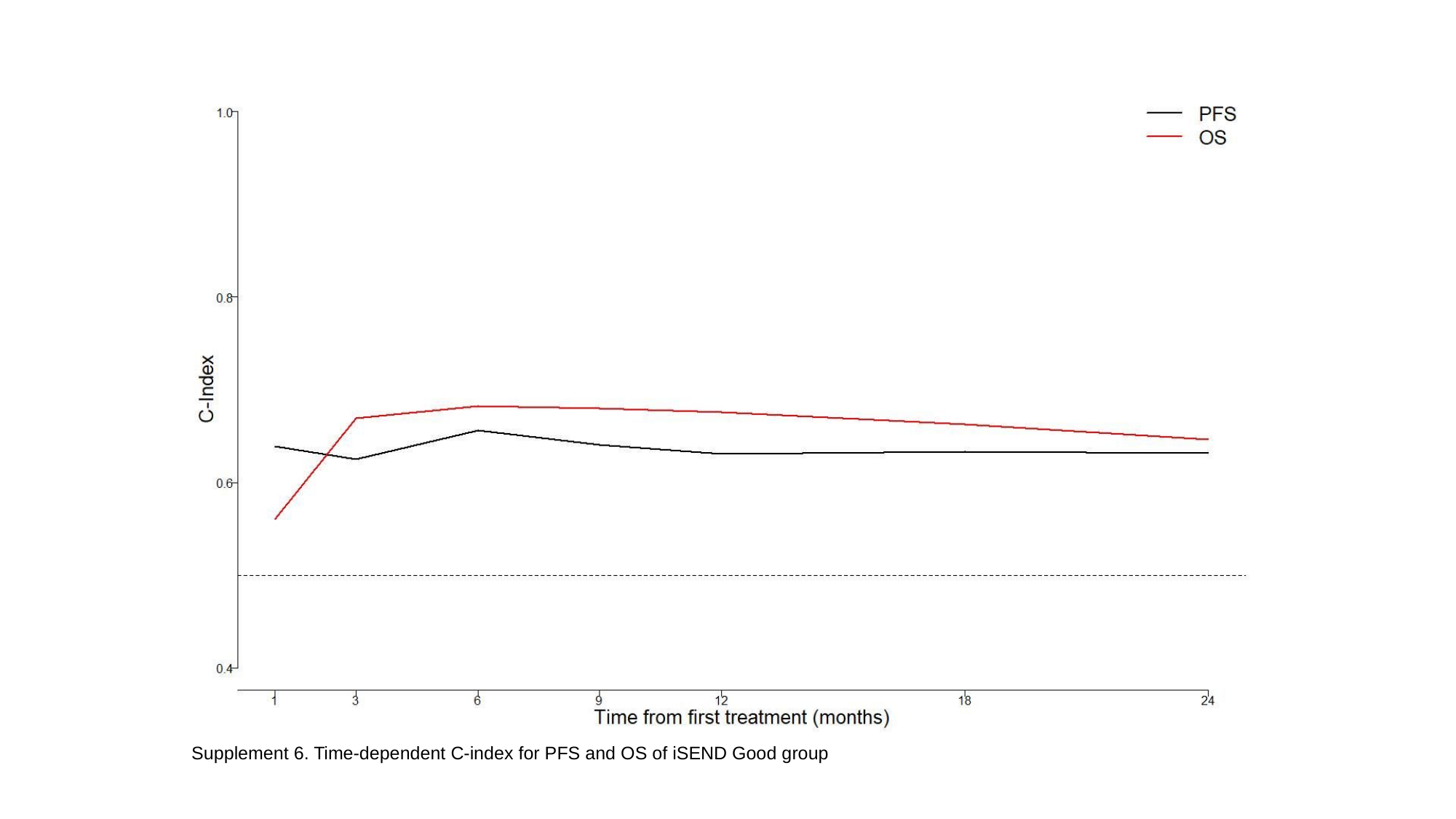

Supplement 6. Time-dependent C-index for PFS and OS of iSEND Good group

## Slide 14
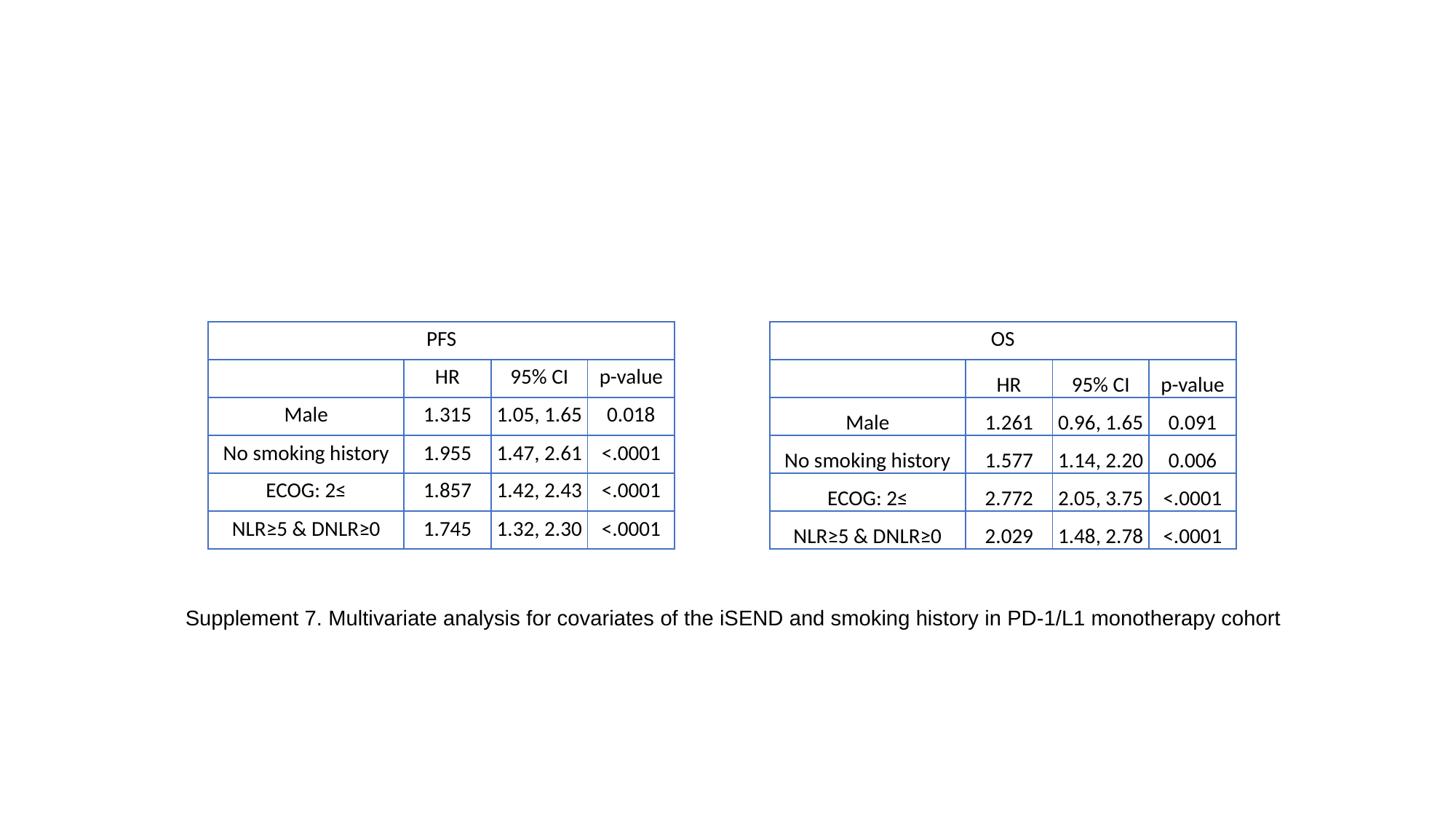

| PFS | | | |
| --- | --- | --- | --- |
| | HR | 95% CI | p-value |
| Male | 1.315 | 1.05, 1.65 | 0.018 |
| No smoking history | 1.955 | 1.47, 2.61 | <.0001 |
| ECOG: 2≤ | 1.857 | 1.42, 2.43 | <.0001 |
| NLR≥5 & DNLR≥0 | 1.745 | 1.32, 2.30 | <.0001 |
| OS | | | |
| --- | --- | --- | --- |
| | HR | 95% CI | p-value |
| Male | 1.261 | 0.96, 1.65 | 0.091 |
| No smoking history | 1.577 | 1.14, 2.20 | 0.006 |
| ECOG: 2≤ | 2.772 | 2.05, 3.75 | <.0001 |
| NLR≥5 & DNLR≥0 | 2.029 | 1.48, 2.78 | <.0001 |
Supplement 7. Multivariate analysis for covariates of the iSEND and smoking history in PD-1/L1 monotherapy cohort

## Slide 15
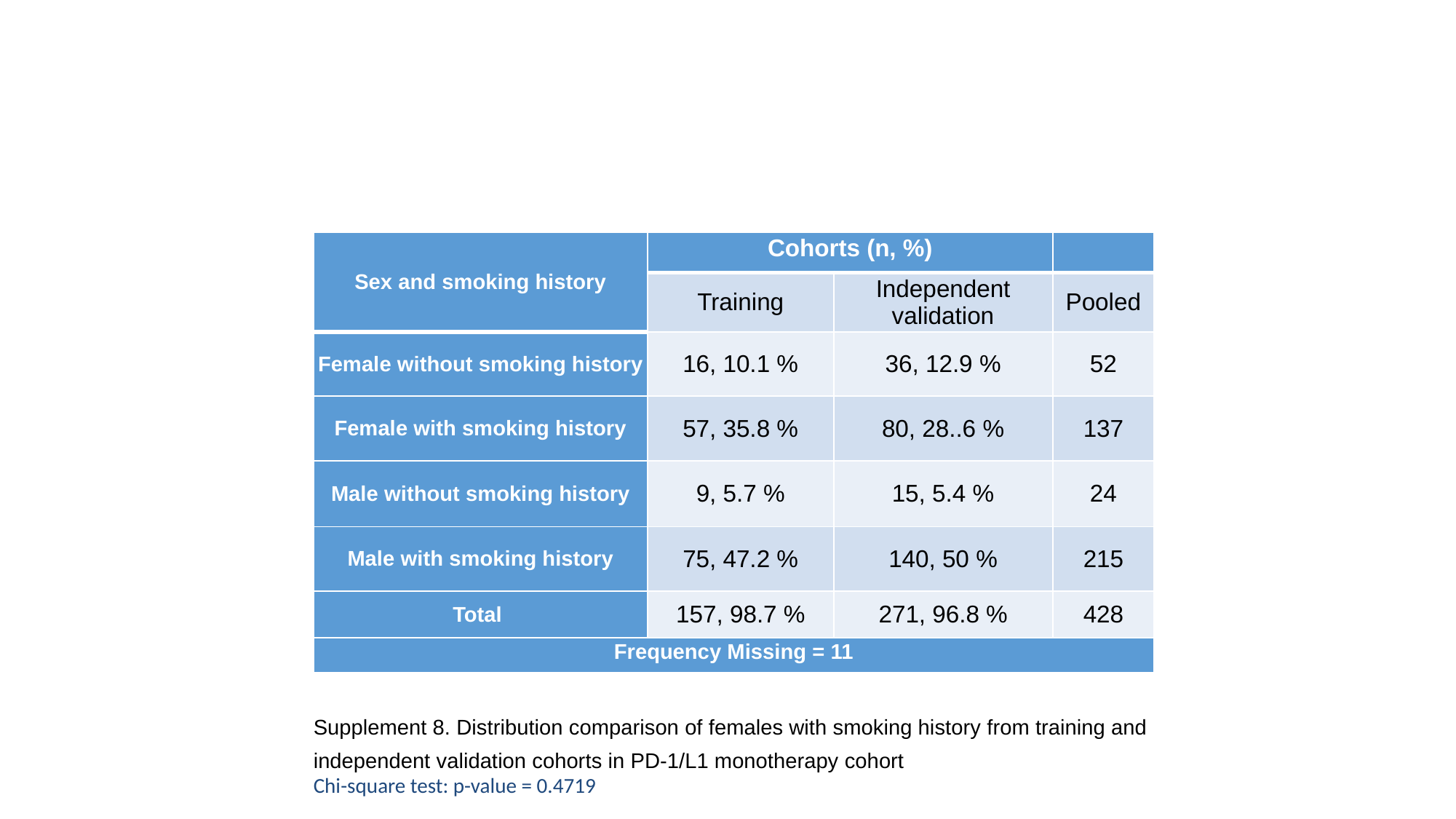

| Sex and smoking history | Cohorts (n, %) | | |
| --- | --- | --- | --- |
| | Training | Independent validation | Pooled |
| Female without smoking history | 16, 10.1 % | 36, 12.9 % | 52 |
| Female with smoking history | 57, 35.8 % | 80, 28..6 % | 137 |
| Male without smoking history | 9, 5.7 % | 15, 5.4 % | 24 |
| Male with smoking history | 75, 47.2 % | 140, 50 % | 215 |
| Total | 157, 98.7 % | 271, 96.8 % | 428 |
| Frequency Missing = 11 | | | |
Supplement 8. Distribution comparison of females with smoking history from training and
independent validation cohorts in PD-1/L1 monotherapy cohort
Chi-square test: p-value = 0.4719

## Slide 16
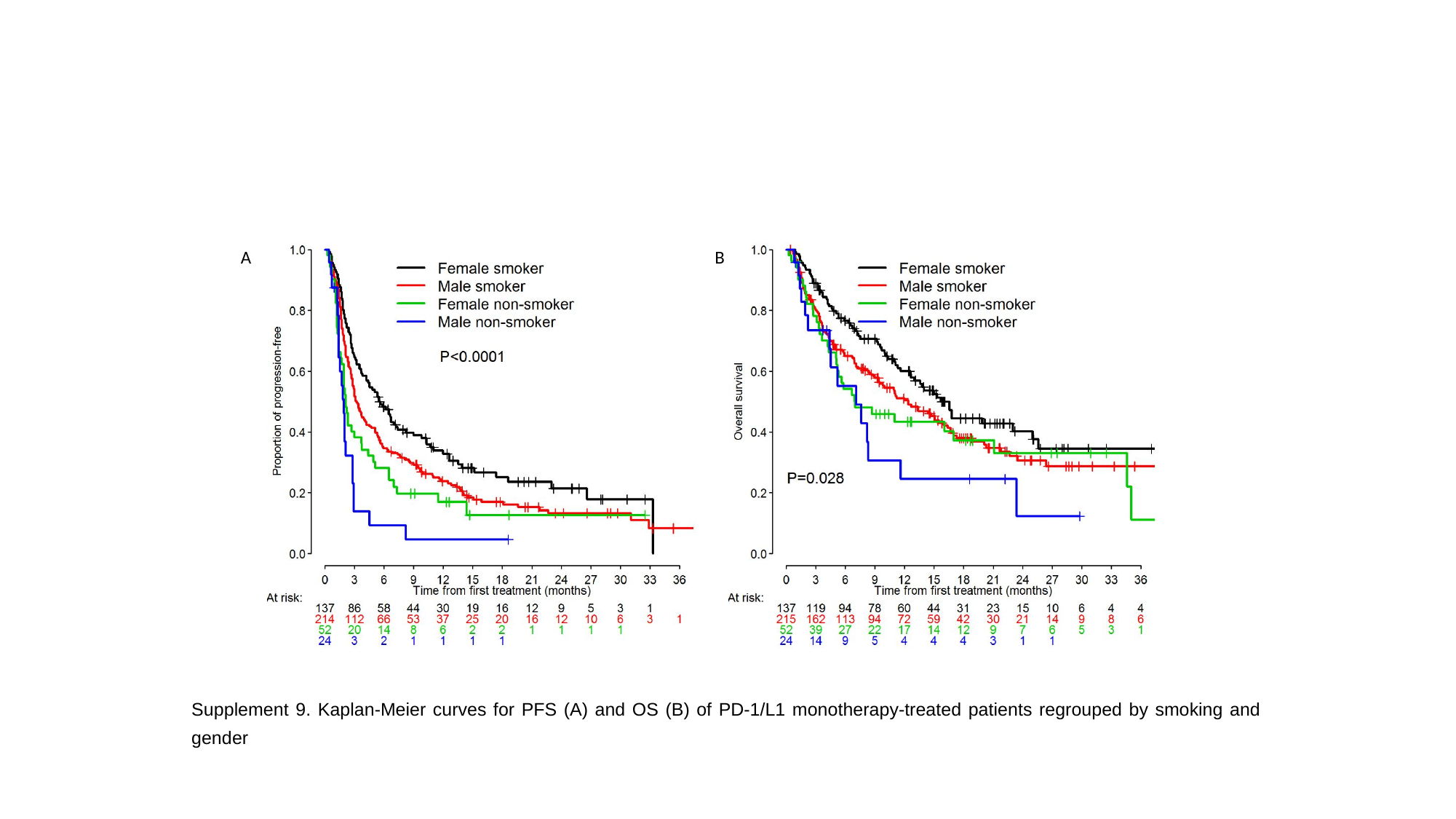

A
B
Supplement 9. Kaplan-Meier curves for PFS (A) and OS (B) of PD-1/L1 monotherapy-treated patients regrouped by smoking and gender
